# Microbial and Antibiotic Susceptibility Profile among Isolates of Clinical Samples of Cancer Patients Admitted in the Intensive Care Unit at Regional Tertiary Care Cancer Center: A Retrospective Observational Study

**DOI:** 10.5005/jp-journals-10071-23119

**Published:** 2019-02

**Authors:** Vishnu Kumar Garg, Seema Mishra, Nishkarsh Gupta, Rakesh Garg, Bharti Sachidanand, Kumar Vinod, Hitender Gautam, Arti Kapil, Sushma Bhatnagar

**Affiliations:** 1-3 Department of Onco-Anaesthesia and Palliative Medicine, Dr BR Ambedkar, IRCH, All India Institute of Medical Sciences, New Delhi, India; 4-9 Department of Microbiology, All India Institute of Medical Sciences, New Delhi, India

**Keywords:** Antibiotics, Antibiotic susceptibility, Cancer, Culture, Multidrug resistance

## Abstract

Cancer patients in intensive care unit (ICU) are vulnerable for developing multidrug resistant nosocomial infections. The antimicrobial resistance due to inappropriate use of antibiotics results in significant morbidity and mortality in these cancer patients. The present retrospective study was done to describe the antimicrobial sensitivity pattern of common organisms in isolates of clinical samples of patients admitted in ICU at our tertiary care cancer center.

**Materials and methods:**

The study was carried out at ICU of a regional tertiary care cancer center for a period of 1 year from October 2016 to September 2017. All clinical samples were collected and processed for culture and antibiotic susceptibility testing were carried out on isolates as per Clinical Laboratory Standard Institute guidelines.

**Results:**

A total of 644 specimens were collected. *Escherichia coli, Acinetobacter* spp., *Klebsiella pneumoniae, Pseudomonas aeruginosa, Staphylococcus aureus* and *Enterococcus* spp. were most commonly encountered. In positive bacterial cultures, majority were Gram-negative isolates (84.14 %). *Klebsiella* was the most common gram-negative isolate (34.78%) and *Enterococcus* spp. were the most common Gram-positive isolates (61.53%). A high level of resistance to various antibiotics was noted among Gram-negative bacteria compared to Gram-positive isolates. Majority of the Gram-negative isolates were sensitive to Imipenem, Meropenem, and Colistin sensitivity among Gram-negative isolates was 100%. Linezolid, Teicoplanin and Vancomycin were most sensitive antimicrobials against the Gram-positive bacteria.

**Conclusion:**

Regular monitoring of the pattern of resistance of bacteriological isolates in cancer patients is critical to develop antibiotic policy to combat these infections and reduce morbidity and mortality.

**How to cite this article:**

Garg VK, Seema M *et al*. Microbial and Antibiotic Susceptibility Profile among Isolates of Clinical Samples of Cancer Patients admitted in the Intensive-care Unit at Regional Tertiary Care Cancer Center: A Retrospective Observational Study. Indian J of Crit Care Med 2019;23(2):67-72.

## INTRODUCTION

**M**ultidrug resistant nosocomial infections are one of the leading causes of morbidity and mortality among hospitalized patients diagnosed with cancer^1,2^. There are usually many risk factors for acquiring infection in these cancer patients, such as long-term catheterization, mucositis due to cytotoxic agents, neutropenia and stem cell transplantation.

Patients in intensive care unit (ICU) are most vulnerable for developing these infections^3^. An ICU patient has five-to sevenfold higher incidence rate of nosocomial infection compared to the general inpatient population and ICU infections contributes to 20-25% of all nosocomial infections in a hospital^4^. This is due to the increasing use of invasive devices, such as mechanical ventilators, monitoring devices, blood and urine catheters, immunosuppressive drugs as well as irrational use of broad-spectrum antibiotic therapy in ICUs^3,5^.

Empirical treatment of infections in the cancer patient is often attempted by administration of broad spectrum or combination antibiotics till the culture and susceptibility results are available.

The in appropriate andirrational use of antibiotics for therapeutic and nontherapeutic use leads to increasing antimicrobial resistance (AMR). In hospital care, this translates into prolonged hospital stay, significant increase in morbidity and mortality and increasing economic burden on the individual and the nation.

The strains of multidrug resistant organisms have become four times worldwide, in recent years^6^. The rising trends of antibiotic resistance in commonly implicated organisms all over the world further enhance the risk of bacterial infections.

The profile of bacteria causing infections and their antibiotic sensitivity pattern vary widely from one geographical region to another as well as from one hospital to another and even among the ICUs within one hospital. Therefore, if the clinician has adequate information of the spectrum of microorganisms and the AMR patterns prevalent in that particular setting, appropriate empiric antibiotic therapy can be started.

The present retrospective study was done to describe the antimicrobial sensitivity pattern of common organisms in isolates of clinical samples of patients admitted in ICU of a tertiary care cancer center in India.

## MATERIALS AND METHODS

We conducted a retrospective, observational study in the ICU of a tertiary care cancer center in India for a period of 1 year from October 2016 to September 2017. The study was conducted after due approval obtained from the Institutional Ethical Committee (IEC-335/01.06.2018).

The study population included patients with various malignancies from medical, surgical and radiation oncology units undergoing treatment in our intensive care unit who had cultures sent for various reasons during study period.

Sample processing, identification of organisms to the species level and antimicrobial susceptibility tests were carried out as per Clinical and Laboratory Standards Institute (CLSI) guidelines, 2016^7^. Antimicrobial sensitivity patterns of respective organisms were studied on Mueller Hinton Agar (MHA) media by KirbyBauer's disk diffusion method^8^. Commercially available discs (HiMedia Laboratories, Mumbai, Maharashtra, India) were used and placed on the surface of the inoculated media and then incubated overnight. Zones of inhibition were measured the next day and were correlated with CLSI interpretive breakpoints to characterize them as sensitive, intermediate, and resistant. For drugs, such as colistin, for which CLSI breakpoints are not available, sensitivity were determined by MIC (minimum inhibitory concentration) method, with E-test strips in accordance with the European Committee on Antimicrobial Susceptibility Testing (EUCAST) guidelines^9^.

Antibiotics to be tested and reported for sensitivity against Gram-negative bacteria were ampicillin (10 μg), gentamicin (10 μg), amikacin (30 μg), amoxycillin-clavulanic acid (20/10 μg), cefotaxime (30 μg), ceftriaxone (30 μg), ceftazidime (30 μg), cefoperazonesulbactam (75/25 μg), ciprofloxacin (5 μg), levofloxacin (5 μg), norfloxacin (5 μg), imipenem (10 μg), meropenem (10 μg), and colistin. For Gram-positive organisms, the antibiotics to be tested and reported were gentamicin (10 μg), erythromycin (15 μg), ciprofloxacin (5 μg), oxacillin (1 μg), ampicillin (10 μg), clindamycin (2 μg), vancomycin (30 μg), co-trimoxazole (1.25/23.75 Mg), doxycycline (30 μg), teicoplanin (30 μg), and linezolid (30 μg).

The data regarding culture and sensitivity of the organisms isolated from different sources were retrieved from the electronic medical records.

### Statistical Analysis

Categorical variables were described as frequency and percentage, and continuous variables as median and interquartile range. For continuous variables, mean values were compared using two sample t-tests for independent samples. Differences in proportions were compared using a Chi-square test or Fisher's exact test, as appropriate. *p* value of <0.05 was considered statistically significant. The analyses were performed using SPSS software.

## RESULTS

All samples were collected as per standard institutional protocol ensuring complete asepsis during collection and handling.

The culture reports of 644 samples from medical oncology (425), surgical oncology (152) and radiation oncology (67) collected during the period from October 2016 to September 2017 were retrospectively analyzed.

Out of these 644 samples, 317 were blood samples, 148 were urine samples, 92 were tracheal samples, 44 were pus samples and 44 were other (sputum, stool, pleural, bronchoalveolar lavage) samples (Table 1).

A total of 31.33% (107/644) samples were positive for growth of organisms. In this positive culture samples, 76.63% (82/107) were bacterial and 23.3% (25/107) were fungal. In the positive bacterial cultures, 84.14% (69/82) were Gram-negative and 15.86% (13/82) were Gram-positive (Tables 2 and 3).

Among the Gram-negatives, the most prevalent organisms isolated were *Klebsiella* (24; 34.78%) followed by *Pseudomonas* (15; 21.73%), *Acinetobacter* spp. (14; 20.28%) and *Escherichia coli* (13; 18.84%). Among the Gram-positives, the most prevalent organisms isolated were *Enterococcus* spp. (8; 61.53%) followed by *Staphylococcus aureus* (5; 38.46%) (Tables 2 and 3).

**Fig. 1:**
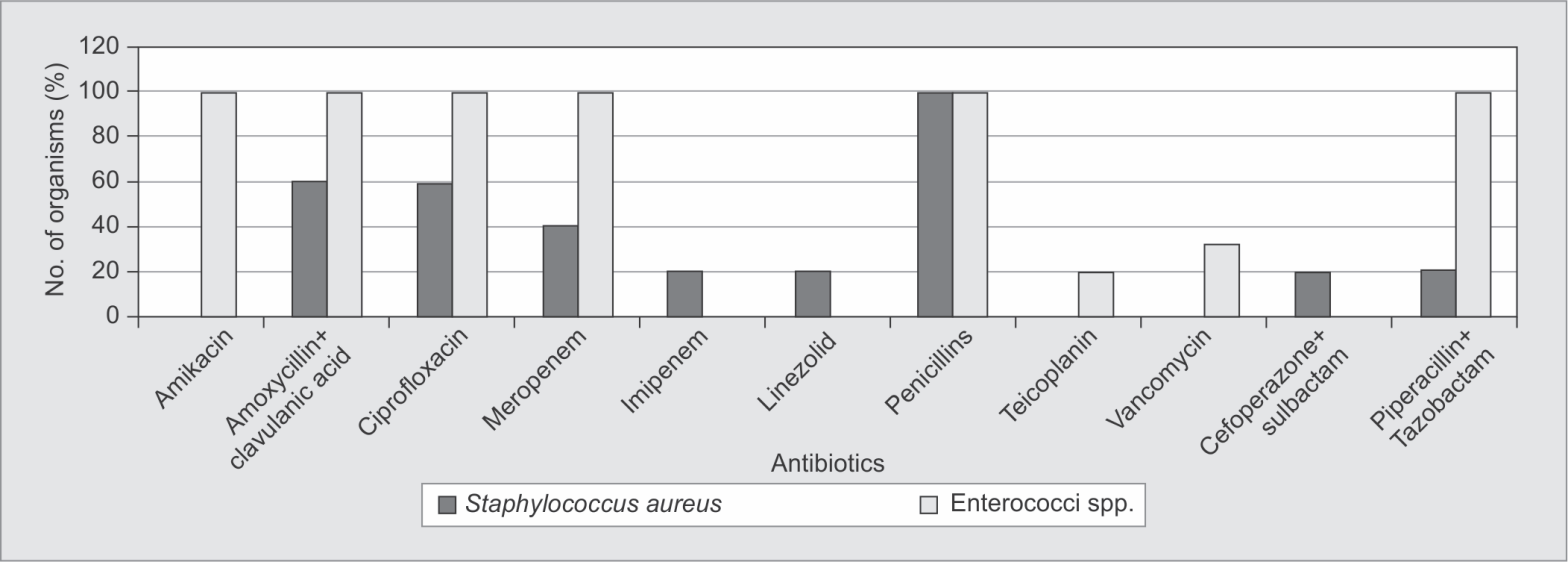
Resistance patterns of Gram-positive organisms

*Staphylococcus* spp. were sensitive to vancomycin, amikacin and Linezolid and all strains were resistant to penicillins. All strains of *Enterococcus* spp. were sensitive to linezolid and resistant to penicillins and ciprofloxacin (Fig. 1).

**Fig. 2:**
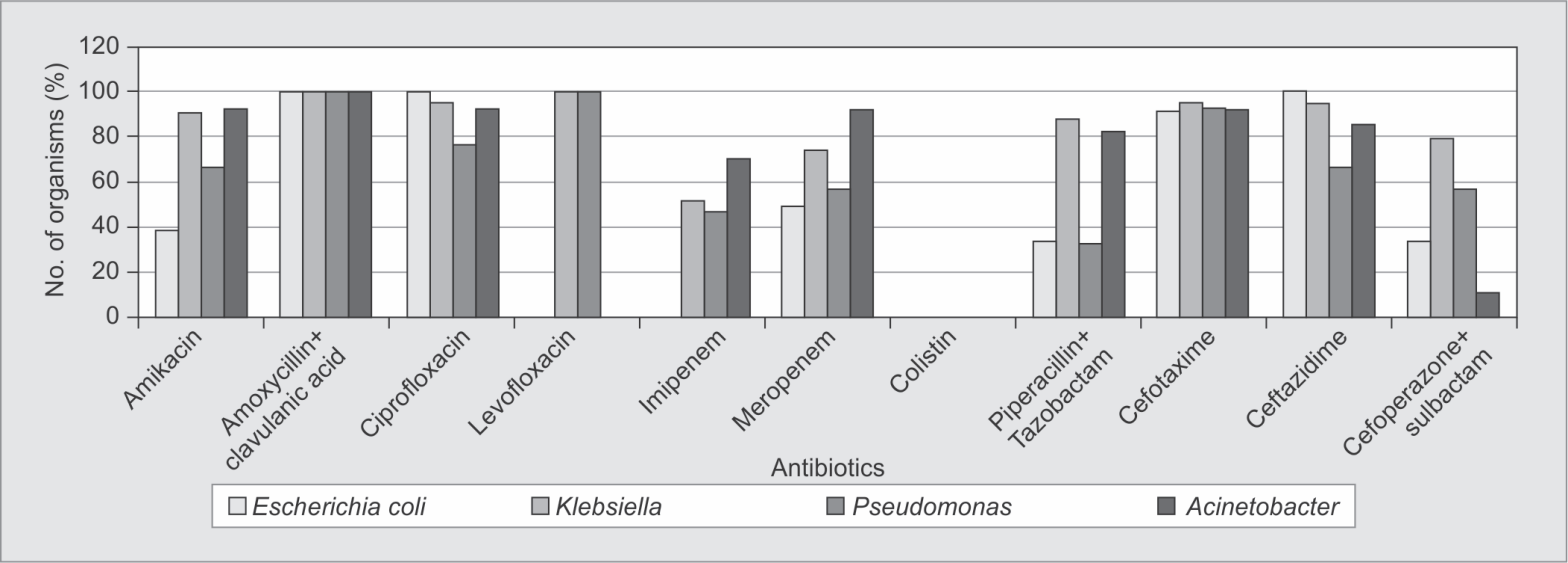
Resistance patterns of Gram-negative organisms

Gram-negative organisms were all susceptible to colistin (100%) and resistant in varying degrees to ciprofloxacin and ceftazidime. Susceptibility to levofloxacin was different for these organisms.*Klebsiella* and *Pseudomonas* spp. were resistant whereas *E. coli* and *Acinetobacter* spp were sensitive to levofloxacin (Fig. 2).

**Table 1:** Distribution of samples taken from patients treated at various oncology department

*Sample*	*Medical oncology*	*Radiation*	*oncology*	*Surgical oncology*	*Total*
Blood	226	37		54	317
Urine	98	16		34	148
Tracheal	57	11		24	92
Sputum	13	2		2	17
Stool	7				7
Pus/Wound	9	1		34	44
Pleural	8			4	12
BAL	7				7
Total	425	67		152	644

BAL: Bronchoalveolar lavage

**Table 2:** Organism profiles in patients treated at various oncology department

*Organism*	*Medical oncology*	*Radiation oncology*	*Surgical oncology*	*Total*
Gram-positive				
*Staphylococcus aureus*		1	4	5
*Enterococcus*	4	1	3	8
Gram-negative				
*Escherichia coli*	8		5	13
*Klebsiella*	17	1	6	24
*Pseudomonas*	11	1	3	15
*Acinetobacter*	7	1	6	14
*Citrobacter*			1	1
*Burkholderia*	2			2
Total	49	5	28	82

**Table 3:** Organism profiles among various samples taken from patients

*Organism*	*Blood*	*Urine*	*Tracheal*	*Sputum*	*Stool*	*Pus/ wound*	*Pleural*	*BAL*	*Total*
Gram-positive									
*Staphylococcus aureus*			1			4			5
*Enterococcus*	6	1				1			8
Gram-negative									
*Escherichia coli*	2	2		2		7			13
*Klebsiella*	9	1	3	7		3		1	24
*Pseudomonas*	4	1	4	3		2		1	15
*Acinetobacter*	1		9	2		1		1	14
*Proteus*									0
*Citrobacter*						1			1
*Burkholderia*	1							1	2
*Fungal*		25							25

BAL: Bronchoalveolar lavage

The antimicrobial sensitivity of organisms isolated from blood is shown in Figures 3A and B. Major Gram-positive organisms were sensitive to antibiotics, such as vancomycin, teicoplanin and linezolid. Gram-negative isolates in the blood had about 40-55% sensitivity to third generation cephalosporins. Among the β-lactam-β-lactamase inhibitor combination that is piperacillin and tazobactam [Pip-Taz], 67% of the *E. coli* isolates were sensitive as compared to only 15% and 40% of *Klebsiella* and *Pseudomonas*, respectively. All Gram-negative isolates were 100% sensitive to colistin.

**Fig. 3A:**
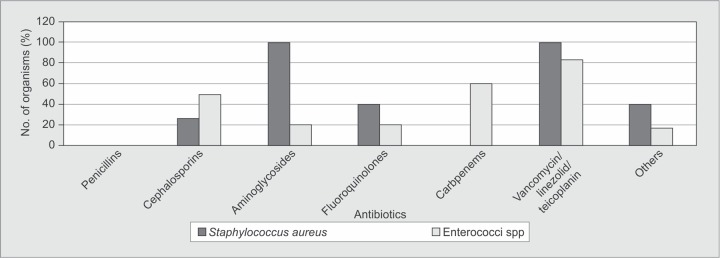
Common Gram-positive isolates sensitivity in blood

**Fig. 3B:**
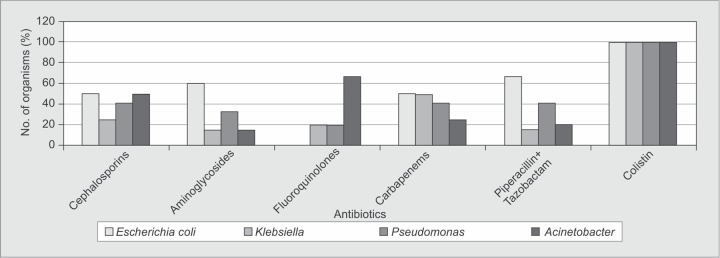
Common Gram-negative isolates sensitivity in blood

**Fig. 4A:**
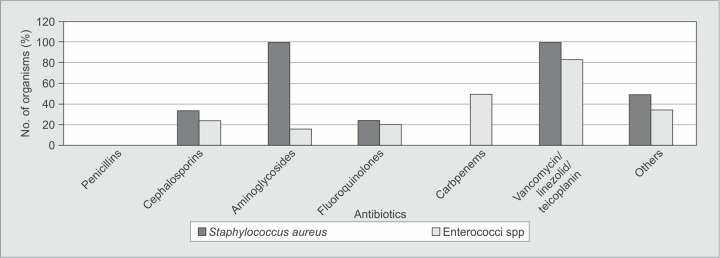
Common Gram-positive isolates sensitivity in respiratory isolates

**Fig. 4B:**
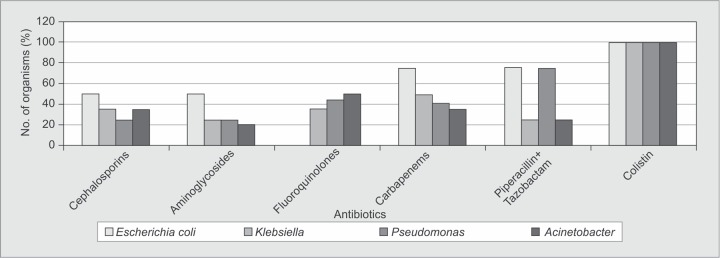
Common Gram-negative isolates sensitivity in respiratory isolates

The antimicrobial sensitivity of organisms isolated from respiratory secretions is shown in Figures 4A and B. *S. aureus* showed no sensitivity to penicillins but 100% sensitive to vancomycin, teicoplanin and linezolid. Almost 100% of various Gram-negative isolates were sensitive to colistin. All the Gram-negative isolates had sensitivity to third generation cephalosporins ranging from 25 to 50%.

## DISCUSSION

Infections are common cause of morbidity and mortality in cancer patients. Various guidelines have recommended early antibiotic therapy on basis of local culture and sensitivity patterns. This prevents unnecessary antibiotics usage and emergence of drug-resistant strains.

In our retrospective study, we have analyzed various cultures sent from patients admitted in our ICU at a tertiary care cancer center. We found that Gram-negative isolates were most common in our setup. This high prevalence of Gram-negative isolates has also been reported by various earlier studies in India in oncology centres^10–13^. Our study findings were also consistent with worldwide results where the predominance of Gram-negative isolates is common as reported in our study. In patients with cancer, the pattern of infections has shifted towards Gram-negative organisms from Gram-positive organisms in the recent years^14–16^. This may be attributed to infrequent use of indwelling catheters, less cytotoxic agents for chemotherapy and decreased use of antibiotic prophylaxis.

In our study, the prevalence of various organisms was as follows:*Klebsiella* (24/82, 29.26%), *Pseudomonas* (15/82, 18.29%), *Acinetobacter* spp. (14/82, 17.07%) and *Enterococcus* spp. (8/82, 9.75%). Similar findings were also observed by Nazneen *etal* ., a study conducted at cancer centre at Aurangabad, Marathwada^12^. Among Gram-negative isolates, *Klebsiella* (34.78%) was the predominant organism followed by *Pseudomonas* (21.73%) in our study. This was in contrast to Singh *et al*. study conducted at cancer center at Delhi where predominant organism was *E. coli* (23.5%) with very low incidence of *Pseudomonas* (6.7%).^10^ This may be due to different antibiotic prescription policies.

In this study, among Gram-positive isolates, *Enterococcus* spp. (61.53%) was most commonly isolated followed by *S. aureus* (38.46 %). This was rather different from prevalence rates in most other studies done in cancer population where predominant Gram-positive organism was *S. aureus*^10,12,17^.

Most of the *E. coli* and *K. pneumoniae* isolates were resistant to the third generation Cephalosporins (cefotaxime/ceftazidime) and β-lactam/β-lactamase inhibitor combinations, such as cefoperazone-sulbactam and Pip-Taz. Similar high rates of resistance of these organisms to the third generation cephalosporins have been noted in a study from Mumbai by Bhat *et al*^11^. and another study from Aurangabad by Nazneen *et al*^12^. However more than 50% of *K. pneumoniae* and *Acinetobacter* isolates were resistant to amikacin, ciprofloxacin.

In our study, the resistance of *Acinetobacter* spp. to aminoglycosides and carbapenems was quite significant. Very high rates of resistance of acinetobacter to ceftazidime (84%), ciprofloxacin (60%) and imipenem (35%) was showed in one study from Delhi, India conducted by Ghosh *et al*.^18^ at a tertiary care cancer center.

The magnitude of antibiotic resistance is fortunately not as high among the Gram-positive compared to Gram-negative organisms. High rates of methicillin resistance [Delhi (35%), Mumbai (42%)]^9,10^ and the emergence of vancomycin intermediate strains of *S. aureus* have also been reported from India. However we did not encounter any resistance to vancomycin among *Staphylococcus*.

This high prevalence of resistant organisms highlights the importance of formulating antibiotic policies based on local antibiotic susceptibility patterns so that arbitrary use of antibiotics is avoided and resistance is kept to a minimum.

### Limitations

Our study has certain limitations. First, it was conducted in a single institution, which may not demonstrate the epidemiology of different centres or different geographical areas. Second, this study provides one-time information about the antibiotic sensitivity which is not sufficient, as the periodic revision of the sensitivity pattern is very essential. Third, being a retrospective study, we did not have the details of the timing of samples sent whether before or on antibiotics, underlying disease and previous antibiotic exposure as these parameters may have different epidemiologic, clinical characteristics, and microbial pattern.

## CONCLUSION

Our study highlights that an antibiogram for ICU patients may help the clinician to understand local susceptibility patterns and help them to make an informed decision about the initial empirical antibiotic.

Regularmonitoring of the pattern of resistance of bacteriological isolates in cancer patients is critical to develop much needed antibiotic policy to combat these infections early. Continuous antibiotic stewardship is required and should be monitored on regular basis to improve outcomes.
